# Stent-Assisted Angioplasty in Spontaneous Bilateral Extracranial Internal Carotid Artery Dissection

**DOI:** 10.3389/fneur.2020.582253

**Published:** 2020-11-13

**Authors:** Mengshi Liao, Xinran Chen, Hongbing Chen, Ying Wang, JinSheng Zeng, Yuhua Fan

**Affiliations:** ^1^Department of Neurology, The First Affiliated Hospital, Sun Yat-sen University, Guangzhou, China; ^2^Guangdong Provincial Key Laboratory for Diagnosis and Treatment of Major Neurological Diseases, National Key Clinical Department and Key Discipline of Neurology, Guangzhou, China

**Keywords:** dissection, intervention, stent, CT perfusion, therapy

## Abstract

Internal carotid artery dissection (ICAD) results from a tear in the intima or rupture of the vasa vasorum with bleeding within the media resulting in separation of the vessel wall layers and a false lumen. It may cause arterial stenosis, occlusion, or dissecting pseudoaneurysm. Currently, the treatment of ICAD is controversial, including drug therapy and endovascular stent implantation. Simultaneous spontaneous dissection of bilateral carotid artery is rarely reported. We reported a 39-year-old-man with bilateral ICAD. Although the long-term durability of endovascular stent remains to be determined, for ICAD failed with active drug treatment and combined with hemodynamic impairment, early endovascular stent should be considered.

## Case Presentation

**Figure 1:**
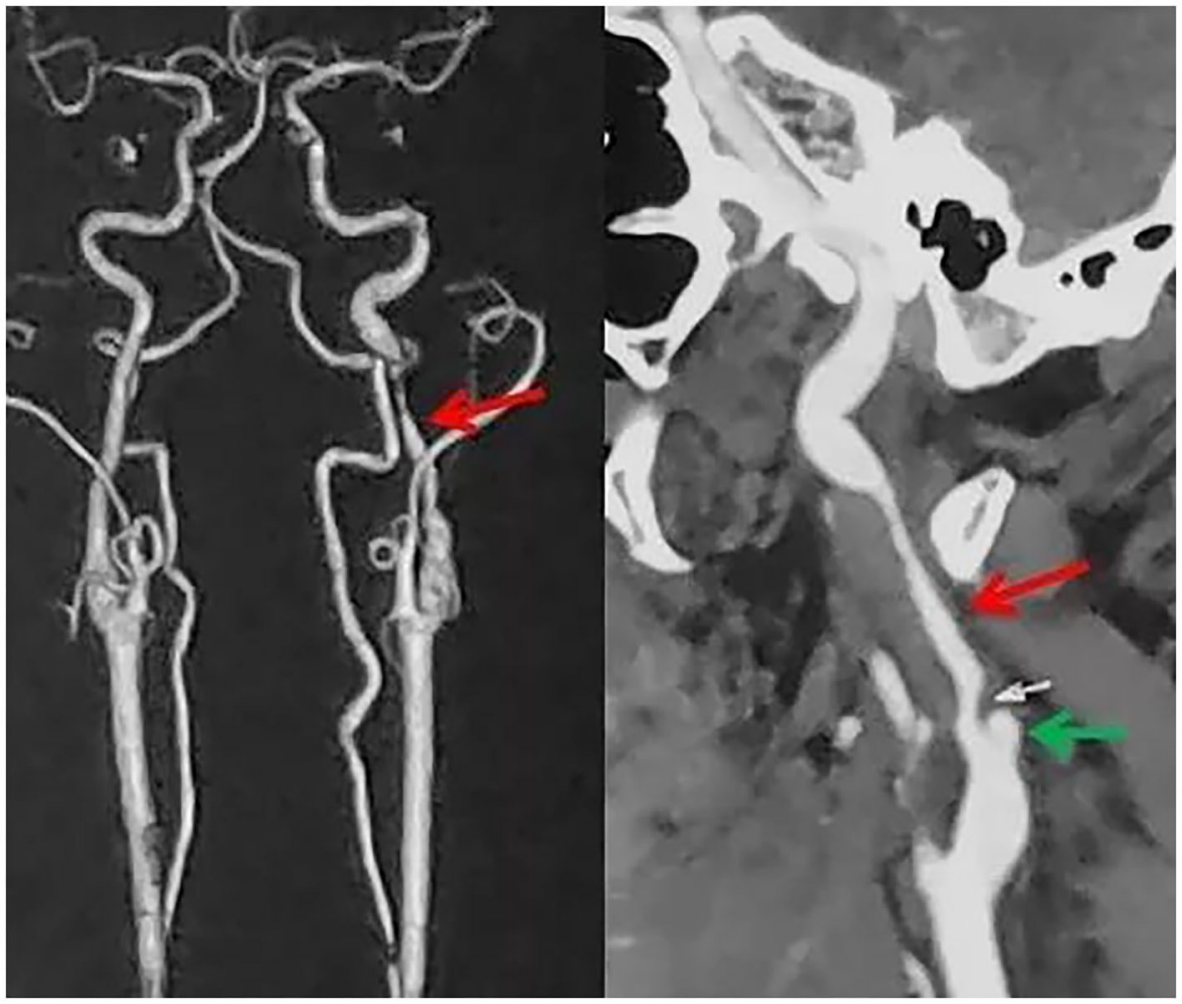
An irregular stenosis was found in the left internal carotid artery (red arrow) with dissecting aneurysm (green arrow). However, there is no clear abnormality on the right internal carotid artery.

**Figure 2:**
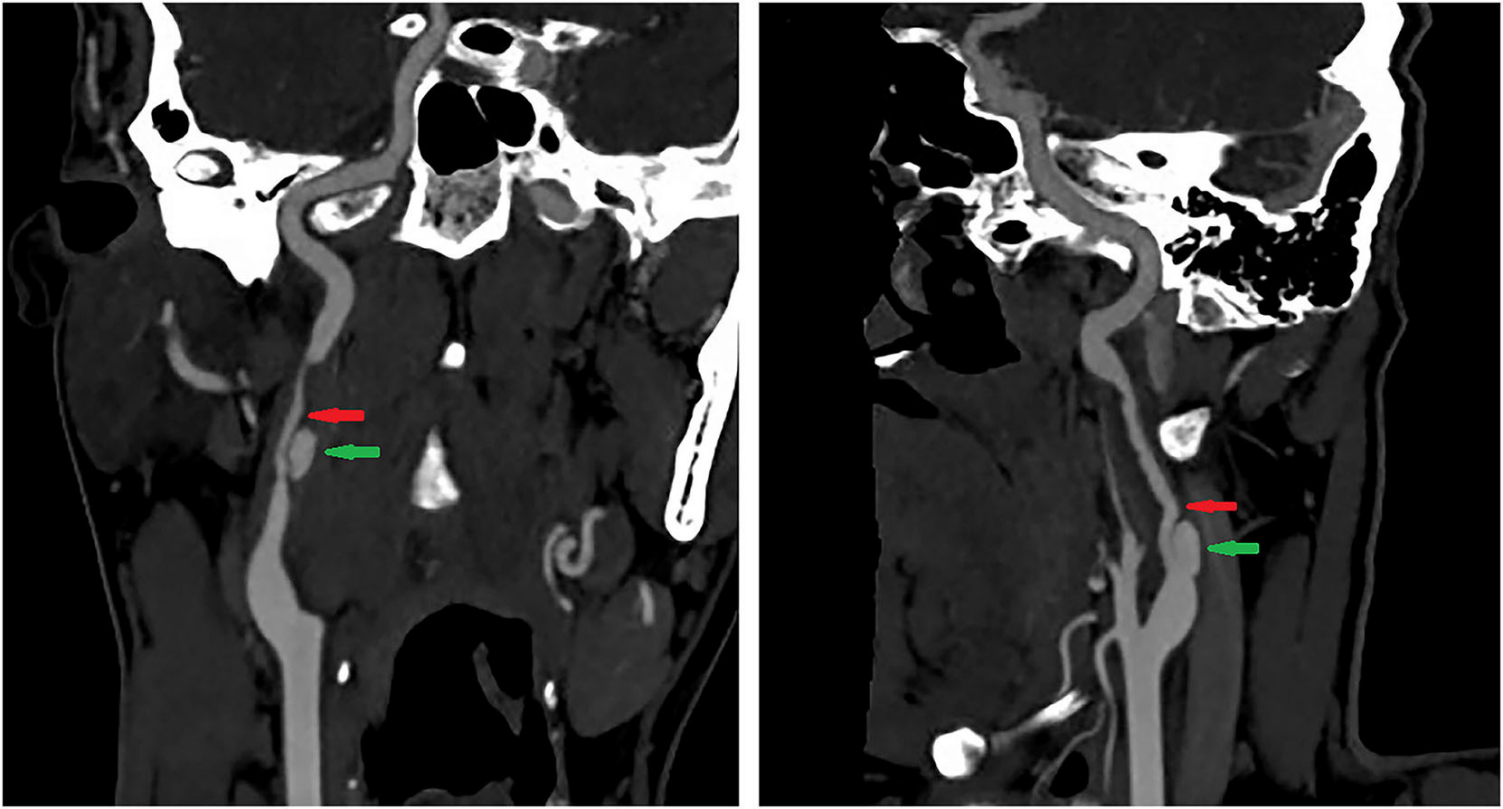
The irregular stenosis of the bilateral internal carotid artery (red arrow) with dissecting aneurysm (green arrow).

**Figure 3:**
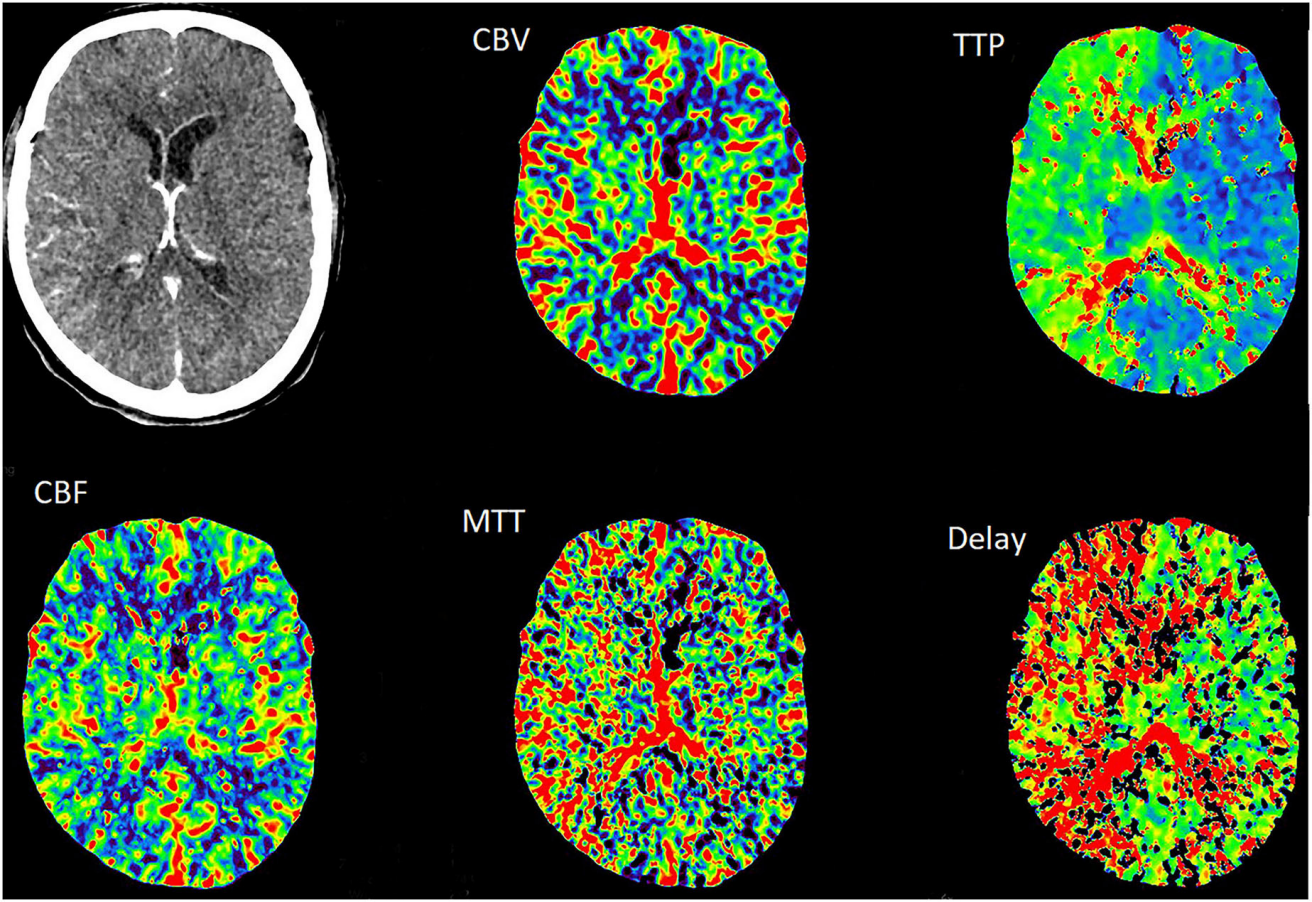
The TTP and MTT in the right cerebral hemisphere were slightly prolonged, whereas the CBF and CBV were normal.

**Figure 4:**
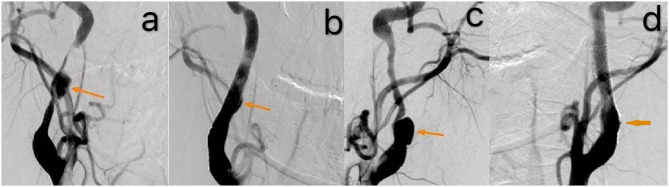
The DSA revealed that the bilateral internal carotid artery was severely narrow with dissecting aneurysm [orange arrow, **(a,c)**]. A smooth blood flow after the operation [orange arrow, **(b,d)**].

A 39-year-old male presented with paroxysmal blurring vision in the left eye and transient weakness in the right limb on August 1, 2019. The symptoms completely disappeared after 1 h. The patient had no history of hypertension, diabetes, heart disease, smoking, and drinking. Furthermore, there was no history of familial genetic disease and similar medical conditions. He was diagnosed as “transient ischemic attack (TIA)” with dissection of the left internal carotid artery (ICA) and was treated with dual-antiplatelet and lipid-lowering drugs from August 3, 2019 on. There was no further neurological symptom from August 3 to August 16. The patient was admitted to our hospital for clarification of the cause of the dissection and further treatment. The patient was treated for the deterioration of imaging findings, with no further clinical symptoms and normal blood pressure of 113/75 mmHg. There was no neck massage or strength exercise recently. The CT angiogram (CTA) of the head performed in the local hospital on August 3 revealed severe stenosis of the left ICA, with the possibility of dissection (Figure 1). The CTA in our hospital on August 15 revealed a bilateral internal carotid dissection artery aneurysm (Figure 2). In addition, the CT perfusion (CTP) imaging revealed that the transit time to the peak (TTP) and mean transit time (MTT) of the right cerebral hemisphere were prolonged, whereas the cerebral blood flow (CBF) and cerebral blood volume (CBV) were basically normal (Figure 3). The digital subtraction angiography (DSA) revealed that the bilateral ICA was severely narrow with dissecting aneurysm (Figure 4).

## Treatment

The patient was treated with aspirin enteric-coated tablets of 100 mg and clopidogrel of 75 mg on August 3. Despite the therapy with dual-antiplatelet, the patient quickly deteriorated from unilateral to bilateral ICA dissection within a short period. Therefore, stenting on the bilateral ICA in local anesthesia was performed on August 16. The stent was placed at the right ICA stenosis to provide two Protégé (EV3, Inc., MN, USA) stents (6 × 40 and 6–8 × 40 mm, respectively). Subsequently, two Protege stents were implanted in the left ICA stenosis. The CTA revealed a smooth blood flow after the operation on the bilateral ICA.

## Outcome and Follow-Up

**Figure 5:**
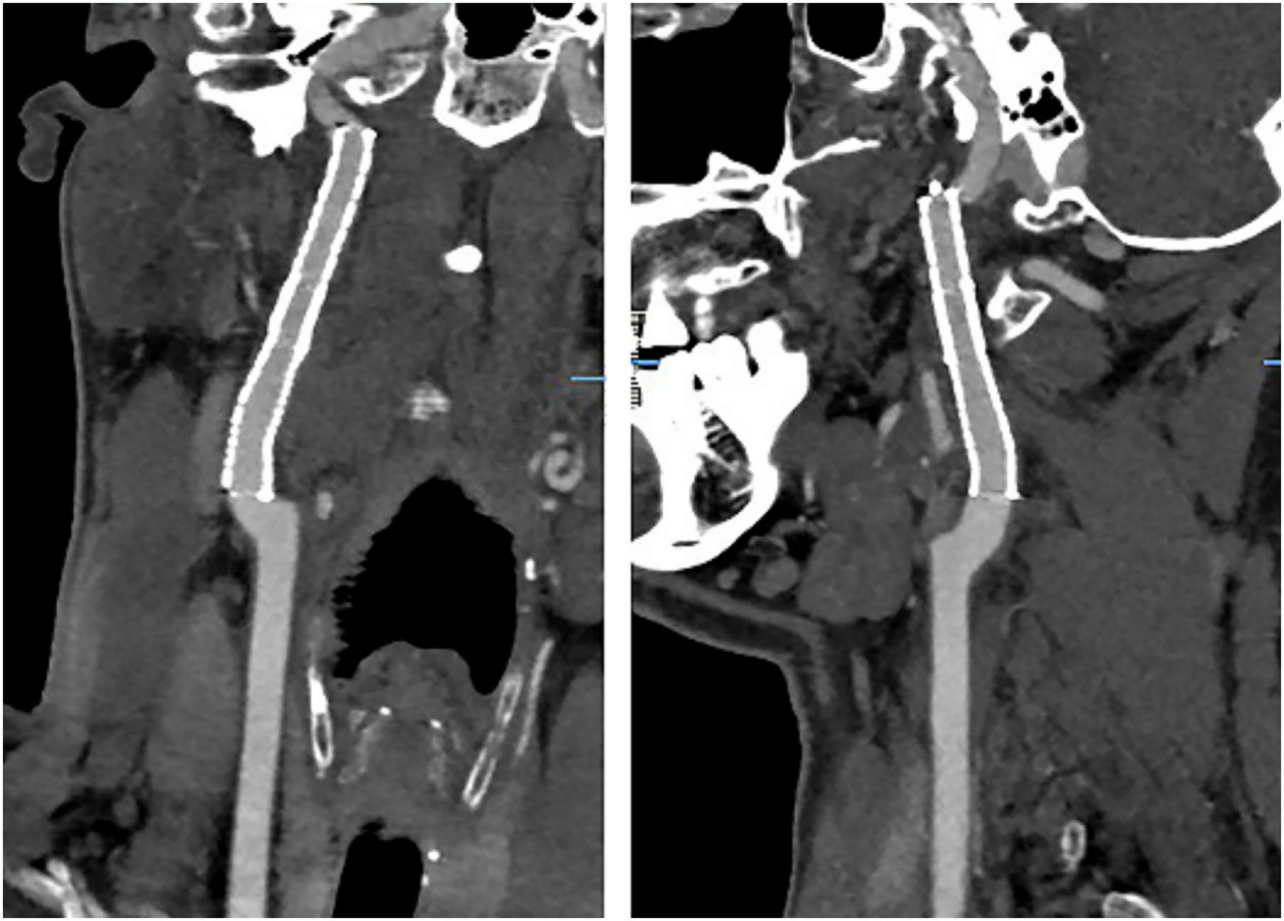
The CTA revealed that the lumen was unobstructed at 3 months after stenting.

**Figure 6:**
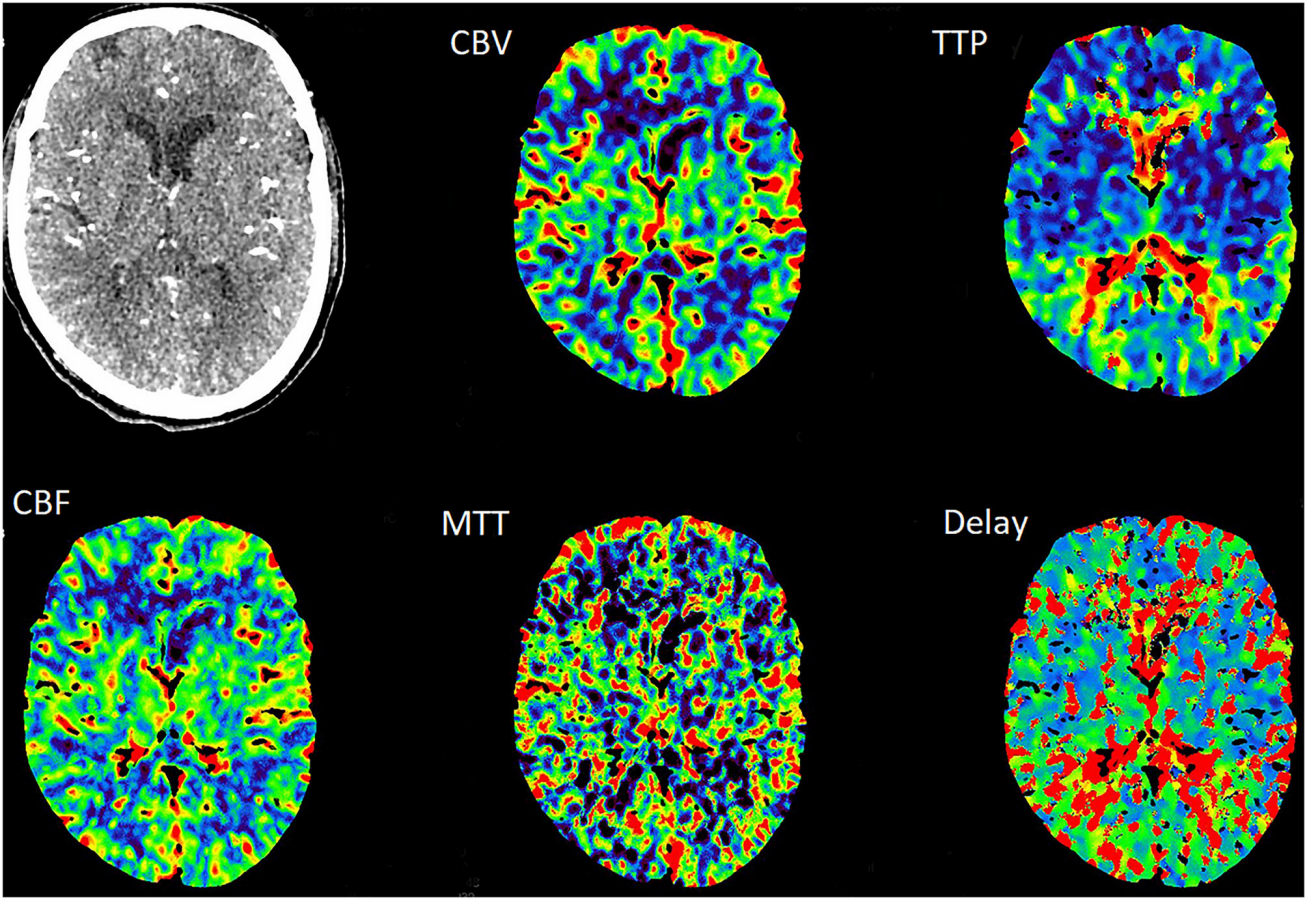
The CTP revealed the normal perfusion of the bilateral hemisphere at 3 months after stenting.

The therapy with dual-antiplatelet was continued for 3 months, and the review of the CTA revealed that the lumen was unobstructed (Figure 5). Furthermore, there was no obvious abnormal perfusion change on the CTP on November 7 (Figure 6). Long-term aspirin was given since then, without recurrence, during the more than 1-year follow-up.

## Discussion

The dissection result from the tear in the intima or rupture of the vasa vasorum with bleeding within the media led to the separation of the vessel wall layers and a false lumen (1). The dissection main risk factors include genetic and environmental factors. In the present case, fibromuscular dysplasia and trauma were not detected, and the patient had no obvious fluctuations in blood pressure levels. The mechanism of dissection involves platelet aggregation and the activation of the coagulation cascade. A number of studies have indicated that aspirin is likely to be as effective as anticoagulation in reducing stroke risk in patients with dissection (1). At present, the treatment of dissection remains controversial, which includes drug therapy and endovascular stent implantation. Hence, it is promising to assess cerebral perfusion during treatment (2). Fanelli et al. reported that stents may be a safe and an effective treatment approach for traumatic bilateral carotid dissections (3). The short-term monitoring of carotid artery stenting (CAS) has exhibited perfect stability of carotid caliber, which shows that endovascular stent placement is a safe and an effective option to restore vessel lumen integrity and prevent stroke (4, 5).

Although few cases on simultaneous bilateral ICA dissection have been reported, the simultaneous treatment of the bilateral dissection has rarely been reported due to various limitations or lack of indications. It has been reported that three cases have successfully performed simultaneous bilateral CAS for dissection, in which the stenosis gradually deteriorated after active antithrombotic drugs (Table 1) (4–6). However, the safety and effectiveness of CAS for simultaneous bilateral dissection have barely been discussed. Therefore, we report a case of bilateral ICA dissection with “TIA” as the clinical manifestation, which was successfully treated with bilateral CAS. The patient was treated with antiplatelet therapy positive and progressed from unilateral to bilateral ICA dissection within a short period and with normal blood pressure of 113/75 mmHg, which possibly shows that the patient's vascular bed was unstable. We considered that antiplatelet therapy may have potentially induced the expansion of the intramural hematoma (7). For the present case, the CTP revealed that the TTP and MTT in the right cerebral hemisphere were slightly prolonged, because the dissection progressed rapidly. Induruwa et al. reported that endovascular treatment is a good alternative to achieve reperfusion in areas of ischemic penumbra caused by carotid artery dissection, where anticoagulation or antiplatelets have failed (8). For the present case, the patient's vascular bed was unstable, which was combined with hemodynamic impairment. The current guidelines had no exact evidence for adopted stent therapy. For the left carotid artery that was symptomatic and with unstable vascular bed, stenting was performed. Meanwhile, CTP showed significant TTP, and MTT delay was found on the right hemisphere with right carotid dissection. Although there were no clinical symptoms, in order to prevent serious disability symptoms in young patients, stenting was also put in at the same time. Hence, the patient was implanted with a bilateral stent at the same time. Furthermore, according to the study conducted by Jiang et al. simultaneous bilateral CAS has no significant adverse events, when compared with unilateral CAS, during the periprocedural period or within 1 year (9). A present literature reported that endovascular intervention had clear advantages but lacks the long-term follow-up study data of CAS (10).

**Table 1:** Bilateral cervical ICA dissection treated with simultaneous bilateral stenting.

**Online time**	**Journal**	**Author**	**Patient image**	**Age**	**Gender**	**Medicine**	**Dissection**	**Treatment**
2003	Interv Neuroradiol.	Sedat et al.	Subarachnoid hemorrhage	53	Male	None	Bilateral internal carotid artery	Simultaneous bilateral carotid artery stenting
2010	J Vasc Surg	Keilani et al.	Ischemic stroke in the left frontal and occipital	52	Female	Antithrombotic drugs for 9 days	Bilateral internal carotid and vertebral artery	Simultaneous bilateral carotid artery stenting
2019	NMC Case Rep	Ishigamet al.	Cerebral infarction in bilateral white matter	46	Female	Dual- antiplatelet therapy for 2 days	Bilateral internal carotid artery	Simultaneous bilateral carotid artery stenting

We report a case that was successfully treated with simultaneous bilateral CAS of the bilateral ICA dissection, without deterioration events during the 1-year follow-up. The long-term durability of the endovascular stent remains to be determined.

## Ethics Statement

Ethical review and approval was not required for the study on human participants in accordance with the local legislation and institutional requirements. The patients/participants provided their written informed consent to participate in this study. Written informed consent was obtained from the individual(s) for the publication of any potentially identifiable images or data included in this article.

## Author Contributions

ML: manuscript conception, design, writing and editing, and clinical care of the patient. XC and HC: manuscript design, final review of the article, and clinical care of the patient. YW and JZ: critical review of the manuscript and clinical care of the patient. YF: manuscript conception, design, critical review of the manuscript, and clinical care of the patient. All authors contributed to the article and approved the submitted version.

## Conflict of Interest

The authors declare that the research was conducted in the absence of any commercial or financial relationships that could be construed as a potential conflict of interest.

## Abbreviations

DWI: diffusion-weighted imaging
TIA: transient ischemic attack
CTA: CT angiogram
CTP: CT perfusion
TTP: transit time to the peak
MTT: mean transit time
CBF: cerebral blood flow
CBV: cerebral blood volume
DSA: digital subtraction angiography
ICA: internal carotid artery
CAS: carotid artery stenting.
